# The Effects of the COVID-19 Pandemic on Trends and Types of Pediatric Burn Injuries: Lessons from a National Burn Center and the Role of Strategic Resource Allocation

**DOI:** 10.3390/life15040544

**Published:** 2025-03-26

**Authors:** Raluca Tatar, Dan Mircea Enescu, Doina Iulia Nacea, Gabriela Viorela Nițescu, Andreea Lescaie, Mihaela Pertea, Petruța Mitrache, Laura Sorina Diaconu

**Affiliations:** 1 Department of Plastic Reconstructive Surgery, “Grigore Alexandrescu” Clinical Emergency Hospital for Children, “Carol Davila” University of Medicine and Pharmacy, 020021 Bucharest, Romania; raluca.tatar@umfcd.ro (R.T.); dan.enescu@umfcd.ro (D.M.E.); petruta.mitrache@drd.umfcd.ro (P.M.); 2 Department of Plastic Reconstructive Surgery and Burns, “Grigore Alexandrescu” Clinical Emergency Hospital for Children, 010621 Bucharest, Romania; 3 Department of Pediatrics, “Carol Davila” University of Medicine and Pharmacy, 020021 Bucharest, Romania; viorela.nitescu@umfcd.ro (G.V.N.); andreea.lescaie@drd.umfcd.ro (A.L.); 4 Pediatric Poison Centre, “Grigore Alexandrescu” Clinical Emergency Hospital for Children, 017443 Bucharest, Romania; 5 Department Plastic Surgery and Reconstructive, Faculty of Medicine, “Grigore T. Popa” University of Medicine and Pharmacy, 700115 Iasi, Romania; mihaela.pertea@umfiasi.ro; 6 Department of Plastic Surgery and Reconstructive Microsurgery, “Sf. Spiridon” Emergency County Hospital, 700111 Iasi, Romania; 7 Department of Internal Medicine III and Gastroenterology, “Carol Davila” University of Medicine and Pharmacy, 020021 Bucharest, Romania; sorina.diaconu@umfcd.ro; 8 Emergency University Hospital of Bucharest, 050098 Bucharest, Romania

**Keywords:** burns, pediatric burns, burn epidemiology, COVID-19 pandemic, resource allocation, healthcare planning

## Abstract

The COVID-19 pandemic had a huge global impact on healthcare systems that affected all medical services, including burn care facilities. This paper analyzes the effects of this medical crisis on pediatric burn injuries by comparing patient data from 2019 (pre-pandemic) and 2020 (during the pandemic) at a national burn center in Romania. The study included, overall, 676 patients, out of which 412 were admitted in 2019. In 2020, the admissions decreased by 35.9% (n = 264). However, moderate and severe burns remained constant and burn severity increased in 2020, with a larger total body surface area affected on average. Surgical management rates and hospital stay duration increased in 2020 from 18% to 39% and from 7 days to 11 days, respectively. Admissions to the intensive care unit and mortality rates remained similar between 2019 and 2020. Scalds were the leading cause of burns in both years; however, in 2020, they affected a larger total body surface area. Contact burns decreased significantly in 2020 from 10.9% to 5.2%, likely due to reduced outdoor activities. The concomitant presence of SARS-CoV-2 infection and burn injuries did not have a negative impact on complication rates, surgical management approaches, or duration of hospitalization. These findings emphasize the need to preserve dedicated burn care human and material resources during global health crises in order to offer access to the best quality of care, thus ensuring optimal patient outcomes, regardless of fluctuations in admission rates.

## 1. Introduction

Burn care requires significant effort from healthcare systems worldwide. The challenge is due to the significant number of cases occurring each year, as well as the complex pathophysiological impact that burn injuries have on the human body. This involves systemic inflammatory reactions, cardio-circulatory and respiratory distress, severe metabolic alterations, and, in case of survival, a high risk for long-term sequelae [1,2,3]. The successful management of such difficult patients can only be achieved by multidisciplinary teams working in highly specialized burn centers or units providing access to extensive resources [4].

When burns occur in the pediatric population, which is traditionally more prone to these injuries, medical professionals must face additional challenges. The higher severity of these cases can be attributed to thinner skin and deeper wounds [5], as well as increased fluid loss to skin surface ratios. Moreover, regardless of age, burn patients have an extremely delicate immune status due to the burn itself, which makes these cases more exposed to infections and sepsis, placing them at a higher risk of developing more extensive and severe forms of disease upon interacting with various microbial agents [6,7,8,9].

The onset of the COVID-19 epidemic in Wuhan, China, in December 2019 [10], followed by the WHO declaring it a pandemic at the beginning of 2020 [9], forced medical authorities and healthcare providers worldwide to rapidly adapt to this unexpected context [11]. This situation raised serious concerns for burn specialists regarding the potential complications that this virus could add to burn shock. At the same time, in regions with high SARS-CoV-2 infection rates, some burn care facilities, including their staff, were reassigned as ICUs for COVID-19 patients, thus redirecting burn patients to other, sometimes less specialized, hospitals [12,13,14]. This decision implied a risk of suboptimal care for burn cases, so many centers around the world started to share their experience regarding management strategies for burn units [12,15] and concerning shifts in burn epidemiology and treatment. The global trend observed in the initial reports was a significant decrease in emergency room presentations and hospital admissions for burn injuries in adult patients, in comparison with previous year(s) [16,17,18,19,20,21]. This observation can be explained by the fact that, in developed countries, burn accidents occur more commonly in relation to the work environment, with fewer cases happening at home. When we examined pediatric burn cases, the published studies showed a less uniform picture. Some centers reported an increased or equal number of cases [8,18,22,23,24,25,26], while others registered a decrease in emergency room presentations and hospital admissions for children with burns [21,27,28,29,30]. Despite striking differences regarding the number of patients and burn surface area (total body surface area—TBSA), all papers reported a similar age group and etiology distribution and a total length of hospital stay comparable with the pre-pandemic data.

Considering all the changes and difficulties brought by the pandemic restrictions, the aim of this paper is to outline the differences registered in pediatric burn epidemiology and treatment strategies, as compared with regular times. Another purpose of our study was to assess how the pandemic context influenced the need for specific burn care materials and human resources.

## 2. Materials and Methods

This paper presents a retrospective analytical study that compares pediatric patients admitted for burns in a tertiary burn center during the COVID-19 pandemic year (2020) with those admitted in the previous year (2019). The inclusion criteria were as follows: age below 18 years, admission to the study center between 1 January 2019 and 31 December 2020, and diagnosis of burn injury. This study adhered to the ethical principles outlined in the Declaration of Helsinki and was approved by the Ethics Committee of the hospital (Approval No. 33486, dated 22 October 2024). All guardians of the patients included in the study provided informed consent for their participation.

To identify eligible patients, the hospital’s electronic health records were screened using the T20.0–T28.4 and T31.00–T31.99 codes of the International Classification of Diseases, 10th Revision, Australian Modification (ICD-10-AM) [31]. All duplicate cases were subsequently eliminated. The collected variables included the date of admission, age, sex, residential area, etiological agent, burn localization (body region), burn extent (percentage representing the affected TBSA), necessity for intensive care, duration of hospitalization in the intensive care unit, and total length of hospital stay (LOS).

Statistical analyses were performed using XL-STAT version 2023.5 (Addinsoft, Paris, France) and VassarStats version SCR-010263 (Vassar College, Poughkeepsie, NY, USA). The patients were stratified according to their year of admission (2019 versus 2020) to facilitate a comparative analysis of their continuous and categorical variables. Continuous variables are expressed as median values with corresponding interquartile ranges (IQRs) and were subjected to a one-way analysis of variance (ANOVA) for comparison. Categorical data are presented as frequencies and percentages, with comparisons conducted using the chi-square test and standardized residuals. Standardized residuals quantify the extent to which an observed value (the one in 2020) deviates from the value established as the baseline (the one in 2019). Statistical significance was established at a *p*-value threshold of <0.05, and at a standard deviation outside the normal distribution of ±1.

## 3. Results

This study included an overall number of 676 patients. In 2019, 412 patients with burn injuries were admitted, whereas in 2020, the number of patients decreased by 35.9%, resulting in a total of 264 patients being admitted. The patient characteristics are presented in Table 1.

The median patient age was similar between 2019 (2.5 years, IQR: 1.33–8) and 2020 (2.29 years, IQR: 1.48–7), with no statistically significant differences across age groups (Table 1).

**Figure 1:**
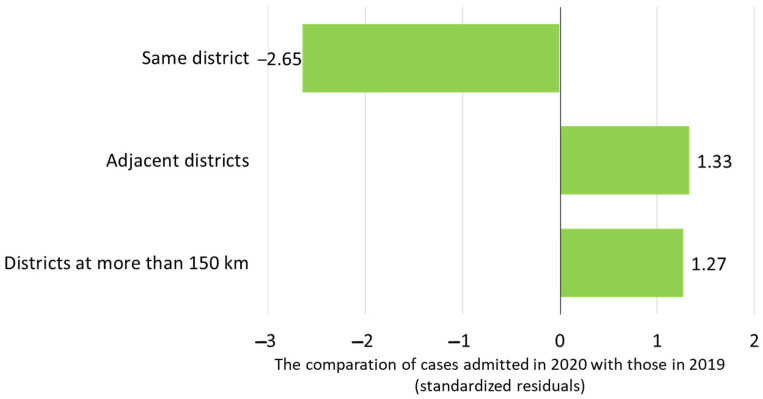
Distribution of patients’ residential areas relative to burn center location for patients admitted in 2020 compared with those admitted in 2019.

The residential location of the patients relative to the burn center was analyzed. In 2019, 38.3% of patients resided in the same district as the burn center, compared to 23.1% in 2020. Patients from adjacent districts amounted to 35.9% in 2019 and 44.3% in 2020. Patients from districts located more than 150 km away accounted for 25.7% in 2019 and 32.6% in 2020. The analysis of patient distribution by residential area revealed that in 2020, there was a smaller proportion of patients from the same district as the burn center, whereas a larger proportion resided in adjacent or distant districts (*p* = 0.0002, Figure 1). However, the time from burn injury to admission was similar between the patients admitted in 2019 and those admitted in 2020 (Table 1).

**Figure 2:**
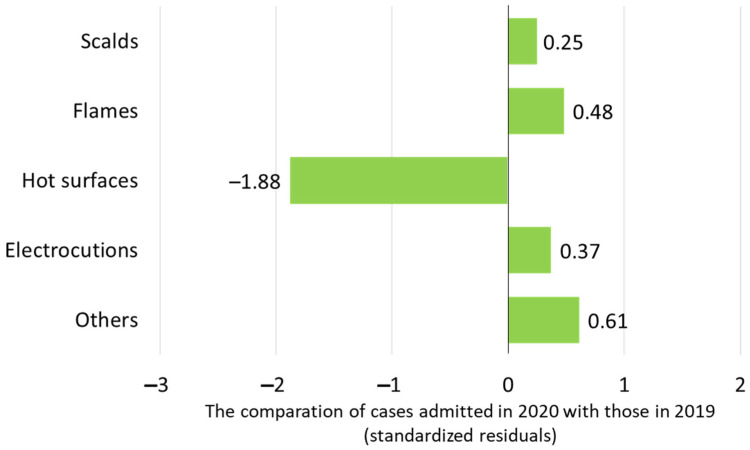
The cause of burns in patients admitted in 2020 compared to those admitted in 2019.

The identified etiological factors for burn injuries were scalds (67.2% in 2019 vs. 69.3% in 2020), flames (18.0% in 2019 vs. 20.1% in 2020), contact with hot surfaces (10.9% in 2019 vs. 5.3% in 2020), electrocutions (2.4% in 2019 vs. 3% in 2020), and others (1.4% in 2019 vs. 2.3% in 2020). The distributions of etiological factors in the pre-pandemic versus the pandemic year revealed a lower proportion of contact with hot surfaces in 2020 compared to 2019 (z-score = −1.88), while the remaining etiological agents were similarly distributed (Figure 2).

Scalds were the most common type of burn injury both in 2019 and 2020; moreover, an analysis of the causal agents for scalds revealed a similar distribution (*p* = 0.16) in both years, with hot water being most frequently incriminated (48.0% vs. 49.7%).

The age distribution of patients by etiologic agents remained similar in 2019 and 2020, with overall lower median ages for scalds and hot surfaces and higher median ages for flames and electrocutions.

**Figure 3:**
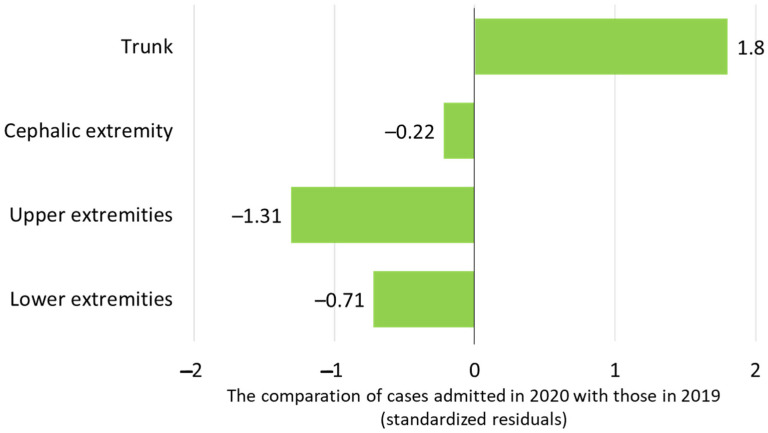
The injured body regions of the patients admitted in 2020 compared with those in 2019.

Burn injuries involved a comparable number of body regions, irrespective of the admission year (*p* = 0.4): one body region was affected in 54.6% of cases in 2019 and in 50% of cases in 2020, and two body regions were affected in 27.9% of cases in 2019 and in 18.9% in 2020, while more than two body regions were affected in 17.5% of cases in 2019 and in 17.4% in 2020. However, when analyzing the distribution of the actual anatomical regions in each year of admission (Figure 3), it was observed that a significantly lower number of patients had burns on the upper extremity (z-score = −1.31), and a significantly higher number of patients had trunk injuries (z-score = +1.8). Moreover, the proportion of patients with burn injuries to the hands was significantly lower in 2020 than in 2019 (15.5% versus 22.6%, z-score = −1.5, *p* = 0.032).

**Figure 4:**
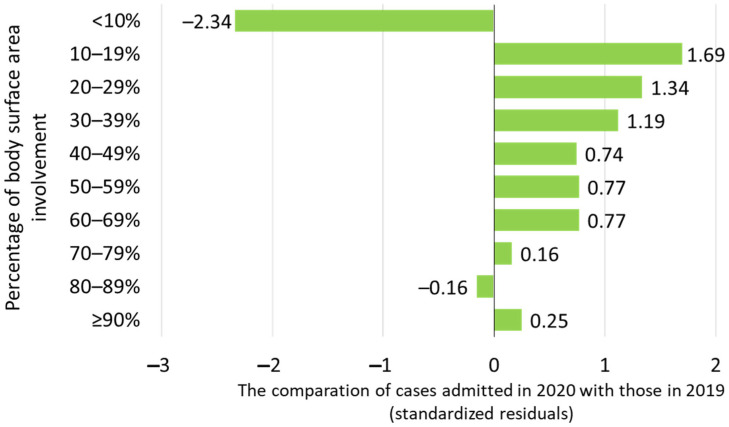
Total TBSA involvement in patients admitted in 2020 compared with those admitted in 2019.

Patients admitted in 2020 had burns with a significantly higher percentage of TBSA than those admitted in 2019 (*p* = 0.0004, Table 1). Moreover, the distribution of patients grouped by the percentage of TBSA exhibited a statistically significant difference in 2020 compared to 2019 (*p* < 0.0001, Figure 4). In 2020, a lower proportion of patients presented with a less than 10% TBSA (z-score = −2.34), while a higher proportion of patients exhibited TBSAs of 10–19% (z-score = +1.69), 20–29% (z-score = +1.34), and 30–39% (z-score = +1.19). Patients with burn injuries affecting 40–49%, 50–59%, 60–69%, 70–79%, 80–89%, and >90% of the body surface area were observed in similar proportions in 2020 and 2019.

Patients with the same percentage of body area involvement in 2019 and 2020 also had similar ages. However, in the subgroup of 80–89% TBSA, there were only two cases in 2019, aged 5.3 and 3.8, and one case in 2020, aged 14, but this observation was not significant (*p* = 0.08).

Scalds admitted in 2020, unlike those admitted in 2019, were associated with a significantly larger burn extension (*p* < 0.0001), with a lower proportion of patients exhibiting a <10% TBSA (z-score = −1.92) and higher proportions of patients with TBSAs of 10–19% (z-score = 1.39), 20–29% (z-score = 1.46), 30–39% (z-score = 3.03), and 40–49% (z-score = 4.70). Burn injuries related to flames, hot surfaces, electrocutions, or other causes showed similar distributions of TBSA between patients admitted in 2019 and those admitted in 2020.

Patients residing in the same district as the burn center in 2020 presented with larger burn extents than in 2019 (*p* < 0.0001). Specifically, there was a lower proportion of patients with a <10% TBSA (z-score = −1.32) and higher proportions of patients with 10–19% (z-score = 2.59) and 50–59% (z-score = 1.36) TBSA. On the other hand, in 2020, fewer patients from adjacent districts presented with <10% (z-score = −1.34), 50–59% (z-score = −2.84), and 60–69% (z-score = −2.84) TBSAs, while the other groups of burn extent were represented similarly to those in 2019. The TBSAs in patients from districts located more than 150 km away from the burn center were also similarly represented in both years.

Intensive care unit admission was required in a higher percentage of patients admitted in 2020 (9.1%, z-score = 1.31) compared to 2019 (5.3%), but without any statistical significance (*p* = 0.08). Moreover, the duration of intensive care treatment was similar between 2019 and 2020 (*p* = 0.72), with median times of 4 days (IQR: 2–5) and 3 days (IQR: 1–5.75), respectively.

Surgical management of burn injuries was required for 18% of patients in 2019 and 39% of patients in 2020, showing a statistically significant higher number of patients who had to undergo surgical procedures in 2020 (*p* = 0.048, z-score = 1.31). The median number of surgical interventions per case was one, both in 2019 and 2020 (IQR: 1–2, *p* = 0.9).

The overall length of hospital stay was significantly higher in 2020 than in 2019 (*p* = 0.0003), with a median of 7 days (IQR: 2–14) in 2019 and a median of 11 days (IQR: 6–18) in 2020.

The mortality rates were 0.97% (n = 4) in 2019 and 0.75% (n = 2) in 2020, highlighting comparable rates between the two assessed years (*p* = 1).

Among the 264 patients admitted in 2020, there was a very small rate of SARS-CoV-2 infection, which accounted for 1.5% (n = 4) of cases. The burn severity in these patients varied from mild to extremely severe (the TBSA ranged from 7% to 90%), having been equally caused by scalding and flame. None of the patients had respiratory distress, nor did they require mechanical ventilation because of COVID-19. The extent of required medical and surgical care, as well as LOS, could be correlated only with the burn severity and not with the presence of the coronavirus, proving that this new viral infection did not have a negative impact on the patients’ outcome; this conclusion is supported by the fact that there were no fatalities in this subgroup.

## 4. Discussion

The COVID-19 pandemic had a huge global impact on healthcare systems, affecting all medical services, including burn care facilities. On the one hand, burn departments had to be reorganized according to the new infectious risks, especially separating the ICU ward, aiming to keep burn patients away from those with SARS-CoV-2 infection. On the other hand, it was necessary for everyone to adopt a strategy for dealing with COVID-19-positive burn patients in order to keep offering them the best possible care, while isolating those cases from the COVID-19-negative patients [32].

In our hospital, during the study period, a separate building was designated as the COVID-19 area, where medical and surgical positive patients were treated. The designated building had individual patient rooms, an ICU ward with ventilators, and an operating theatre where all the necessary surgeries were performed, using the appropriate personal protective equipment for all the medical staff. The COVID-19-positive patients in our group were treated there, two of them undergoing surgical excision and grafting of burn areas. The rest of the burn patients were admitted to the burn ward in another building of the same hospital. We have already presented more details about how our department dealt with the restrictions at the beginning of the pandemic in a communication published in December 2020 [33].

As a consequence of the lockdown measures, most burn centers around the world registered a significant decrease in the number of total burn patients and burn admissions, mostly reported for adults [24,30,34,35]. Pediatric burn admissions were very unevenly distributed around the world [8,36,37,38,39]. In our study, a decreasing trend was maintained throughout 2020, when we had 35.9% fewer inpatients than the previous year. However, in other geographic areas, there were reports of an increase in child burns [5,22,25,40,41], explained by the fact that, although parents and children were staying home, remote working kept adults from being constantly aware of what children were doing, thus exposing them to various risks of getting burned [39,42]. Even before the COVID-19 pandemic, the literature described situations where the presence of an adult in a child’s proximity did not ensure proper supervision and a safe environment for preventing pediatric burns [43].

Despite having fewer patients, some of the epidemiological characteristics of burns followed the same distribution pattern as they did in pre-pandemic times. We noticed the same male preponderance and a similar age group distribution, involving mostly children below the age of five. Our findings are also in accordance with the generally known epidemiological features of burns in the pediatric population [44,45,46,47,48]. Considering that our department is the national referral center for pediatric burns in Romania, we assessed the place of residence of our patients in order to see how many came from distant regions of the country. Regardless of the traffic restrictions and isolation regulations, we found that, in 2020, a larger proportion of patients originated from districts beyond the immediate vicinity of the burn center’s location and its surrounding areas, many of them (44.3%) having to cover a distance surpassing 150 km.

These findings were statistically significant and even had an impact on the time of discharge, influencing the total length of hospital stay (LOS) for patients coming from very distant places (sometimes more than 400–500 km). The LOS in 2020 was higher than in 2019 not only because of burn severity, which was higher, as we will further see, but also because we had to keep patients admitted for longer periods since it was difficult to ensure proper remote follow-up upon discharge, and we preferred to have them monitored until complete wound coverage and healing. Another reason for longer hospital stays was to ensure a correct rehabilitation program that was not available elsewhere anymore, also because of pandemic restrictions and shifts in local medical facilities toward SARS-CoV-2-infected patient care [49]. There were also other centers reporting a longer LOS during the pandemic throughout the literature [5,18,50]. We also noticed a shift in the urban to rural ratio between the two years, with more patients coming from the countryside in 2020, probably because the restrictions were less severe there and children had access to wider areas and more potentially harmful situations.

The majority of burns were caused by scalds in both years, as it was previously stated in the literature [51,52] and observed during the pandemic [36,39], and the other etiological agents mostly kept the same proportion. Additionally, the etiological stratification by age groups did not show significant differences in the two years of the study, with scalds being more frequent in infants and toddlers and flame and electrical burns in older children and teenagers. The same distribution was also found in other settings [5,49]. A statistically significant change was noticed in contact burns, which decreased by two-thirds, as was reported as well by other research papers [53]. This situation could be explained by the fact that usually, at least in our experience, these accidents occur in outdoor activities when children touch hot objects, such as grills or candle taps, scenarios that were less frequent in the first year of the pandemic. This was correlated with the smaller number of hand burns that we registered in 2020, since young children usually explore the environment by touching everything with their hands [46].

The analysis of burn severity, as reflected by the percentage of TBSA involved, showed the most important findings regarding resource allocation and the need for specialized staff and facilities. When looking at the mean value of TBSA, we noticed a higher value in 2020, with statistical significance, meaning that we had to deal with more severe cases than in 2019, a finding reported as well by a Spanish study [18]. When looking at the distribution by TBSA percentage groups, we found that the general decrease in admissions was due to the decrease in minor burns (i.e., burns involving less than a 10% TBSA). Nonetheless, the number of moderate and severe cases, those that require highly specialized and complex care, dedicated beds and staff, multidisciplinary teams, and sometimes ICU admission, was similar. Based on this observation, we can state that the incidence of severe burns (i.e., burns that surpass a 20–30% TBSA) has its own dynamics, independent of the pandemic and global medical crisis. Taking this into consideration, and the fact that we registered an increased need for the surgical treatment of our patients during pandemic times, it is of the utmost importance to keep all the necessary centers and personnel available to ensure adequate care. In our case, because the local regulations postponed elective surgeries for several months while maintaining all the structures and staff for emergency cases (including burns), we were able to continue offering the same standard of care for burned children and had a low mortality rate, comparable with that of developed countries [45,48]. It should also be emphasized that, despite the potential risk of getting infected with the SARS-CoV-2 virus, patient transfer from very distant places to our center offered the best chance for optimal care and survival for every single one of them.

Among the changes that came with the pandemic, we noticed that scald burns involved a larger TBSA when compared with the previous period. This situation may be related to parental inability to foresee the consequences of their actions, which put children at risk of getting burned. At the same time, it may be an effect of the “stay at home” and “work from home” new reality, which creates an impression of carefully watching the child while at home. In fact, when the parent is focused on work tasks, their attention cannot be directed to the child, who acts as if they were alone, exploring and endangering themselves [42].

Another important problem raised by the pandemic was the difficulty to reach medical facilities and to obtain access to specialized care [23], a downside that was reflected in a decreased quality of care for patients with different medical conditions other than the SARS-CoV-2 infection [54,55,56]. In our setting, we could strongly state that the situation was different: the time from burn injury to admission was similar to that in the pre-pandemic time, and the quality of care, as previously shown, was also kept at the best of standards. These findings reinforce the importance for the burn care infrastructure and human resources to be maintained unchanged, independently of the other pressures inflicted on the health care system [18], since the incidence of moderate and severe burn cases proved not to be influenced by the pandemic context [39,57]. Any alteration in the established functioning of burn care departments would potentially have a significant negative impact on patient access to qualified services and on the outcome of every single case.

Regarding the concomitant presence of SARS-COV-2 infections in pediatric burn patients, it did not seem to have a significant influence on patient evolution during hospitalization and on the clinical result. As a matter of fact, the COVID-19 disease in children has been described as mostly mild in severity and less frequent than in the adult population [58], albeit serious neurological manifestations have been reported in the pediatric population as well [59]. Although this infection may worsen their general state and lead to a poor outcome in patients with previous comorbidities, it generally has no clinically meaningful impact on children and teenagers that were healthy before the accident [5,60,61]. Although it investigated a significant number of cases, this research has limitations related to the fact that it was a retrospective study that could not thoroughly assess all the particularities related to the moment when the burn injury occurred and the treatment protocol, due to missing data and/or recording and misclassification errors. At the same time, the data that could be assessed regarding the concomitant presence of COVID-19 and burns in children were very limited, so further research should focus on larger patient samples. Thus, more reliable conclusions about the effects of this special medical condition in burn patients could be provided.

## 5. Conclusions

Our study offers a comprehensive insight into the impact the COVID-19 pandemic had on the epidemiology of pediatric burns. It emphasizes the fact that “stay at home” does not necessarily imply “stay away from harm”, because, despite fewer admissions, the number of moderate and severe burns remained stable. A higher proportion of severe cases was registered in 2020, with a greater total body surface area affected, a longer length of hospital stay, and an increased need for surgical treatment. At the same time, this research emphasizes the need to preserve both the human and material resources dedicated to burn care during global health crises in order to ensure access to high-quality care, thus preserving optimal patient outcomes, regardless of fluctuations in admission rates.

## Figures and Tables

**Table 1:** The characteristics of patients admitted in 2019 compared with the ones of the patients admitted in 2020.

Variable		Admitted in 2019 (n = 412)	Admitted in 2020 (n = 264)	*p*-Value
**Sex distribution:**				
	Males, n (%)	267 (64.8)	159 (60.2)	0.29
	Females, n (%)	145 (35.2)	105 (39.8)	
**Age distribution:**				
	Median age, years (IQR)	2.5 (1.33–8)	2.29 (1.48–7)	0.64
	<1 year, n (%)	34 (8.3%)	19 (7.2%)	0.79
	1–4 years, n (%)	237 (57.5%)	159 (60.2%)	
	5–9 years, n (%)	61 (14.8%)	41 (15.5%)	
	10–14 years, n (%)	49 (11.9%)	31 (11.7%)	
	15–18 years, n (%)	31 (7.5%)	14 (5.3%)	
**Residential area distribution:**				
	Urban, n (%)	203 (49.3)	115 (43.6)	0.17
	Rural, n (%)	209 (50.7)	149 (56.4)	
**Residential location of patients relative to the burn center:**				
	Same district, n (%)	158 (38.3%)	61 (23.1%)	0.002
	Adjacent districts, n (%)	148 (35.9%)	117 (44.3%)	
	Districts at more than 150 km, n (%)	106 (25.7%)	86 (32.6%)	
**Time from burn injury to admission:**				
	Median time, hours (IQR)	5 (2–24)	5 (3–18)	0.51
**Etiological agents:**				
	Scalds, n (%)	277 (67.2%)	183 (69.3%)	0.13
	Flames, n (%)	74 (18.0%)	53 (20.1%)	
	Hot surfaces, n (%)	45 (10.9%)	14 (5.3%)	
	Electrocutions, n (%)	10 (2.4%)	8 (3.0%)	
	Other—sun, n (%)	3 (0.7%)	5 (1.9%)	
	Other—chemicals, n (%)	3 (0.7%)	1 (0.4%)	
**Type of involved body regions:**				
	Trunk, n (%)	199 (48.3%)	165 (62.5%)	0.027
	Cephalic extremity, n (%)	73 (17.7%)	50 (18.9%)	
	Upper extremities, n (%)	225 (4.6%)	121 (5.8%)	
	Lower extremities, n (%)	189 (45.9%)	110 (41.7%)	
**Percentage of body surface area involvement** **:**				
	Median percentage, % (IQR)	6 (4–12)	10 (5–20)	0.0004
**Intensive Care Unit admission:**				
	Number of patients (%)	22 (5.3%)	24 (9.1%)	0.08
	Median time, days (IQR)	4 (2–5)	3 (1–5.75)	0.72
**Management type:**				
	Surgical	74 (18.0%)	64 (39.0%)	0.048
	Conservative	338 (82.0%)	200 (61.0%)	
**Overall hospitalization time:**				
	Median time, days (IQR)	7 (2–14)	11 (6–18)	0.0003

## Data Availability

The data that support the findings of this study are available from the corresponding author upon reasonable request due to privacy concerns.
